# Safety and Immunogenicity of a Single Dose of BNT162b2 COVID-19 mRNA Vaccine in a Warfarin-Treated Protein S Deficient Patient: A Case Report and Literature Review

**DOI:** 10.3390/hematolrep14040051

**Published:** 2022-12-05

**Authors:** Cucunawangsih Cucunawangsih, Ratna Sari Wijaya, Nata Pratama Hardjo Lugito, Ivet Suriapranata

**Affiliations:** Faculty of Medicine, Pelita Harapan University, Tangerang 15811, Indonesia

**Keywords:** COVID-19, vaccination, BNT162b2, antibody, protein S deficiency

## Abstract

Patients with protein S (PS) deficiency possibly have a higher risk of developing severe COVID-19 disease. Therefore, vaccination against SARS-CoV-2 infections is recommended for PS-deficient patients. However, there are limited data regarding the safety and immunogenicity of the currently available COVID-19 mRNA vaccine in PS-deficient patients. We report a case of monitoring the antibody response of a 40-year-old female diagnosed with PS deficiency and on warfarin treatment following a single dose of BNT162b2 mRNA vaccine. Antibody against the receptor-binding domain (RBD) of the SARS-CoV-2 spike (S) protein (anti-S) was measured on days 7, 14, and 21 after vaccination. Seroconversion was detected on day 21 but was possibly lower than the anti-S level previously reported in healthy individuals after receiving the first dose of the BNT162b2 mRNA vaccine. There were no local and systemic events reported up to 7 days in this patient after vaccination. This case highlights that the administration of the BNT162b2 vaccine had a favourable safety profile, and the second dose of the vaccine is required to provide the optimal protection against SARS-CoV-2 infection in PS-deficient patients.

## 1. Introduction

Protein S (PS) deficiency is a rare disorder and can be hereditary or acquired [1]. PS deficiency has been suggested to contribute to hyperinflammation and hypercoagulability states and is associated with the severity and mortality outcome of COVID-19 disease [2,3,4]. Thus, vaccination against SARS-CoV-2 is recommended for individuals with PS deficiency. However, there are limited data about the safety and immunogenicity of the COVID-19 mRNA vaccine in PS-deficient individuals. This report evaluates a PS-deficient patient’s safety and antibody response who received a single dose of the BNT162b2 COVID-19 mRNA vaccine.

## 2. Case Description

The research ethics committee of the Medical Faculty of Pelita Harapan University approved this study (No: 137/K-LKJ/ETIK/IV/2021). Informed consent was obtained for the publication of this case report. A 40-year-old Indonesian female with PS deficiency history and on warfarin treatment was diagnosed a few years ago with PS deficiency with a serum protein S level below the normal range of 70–140%, whereas protein C was normal. There was no history of family with thrombophilia or PS deficiency. No thromboembolic events were identified in the patient and her family history. Her BMI is 24.3 kg/m^2^; she is non-smoker and has no co-morbidities. She works as a nurse in the Intensive Coronary Care Unit (ICCU) at Siloam Hospital, Indonesia. She had no prior polymerase chain reaction-confirmed diagnosis of COVID-19.

The total antibodies (combination of IgA, IgM, and IgG) against the receptor-binding domain of the SARS-CoV-2 spike (S) protein (anti-S) level were measured using the Elecsys anti-SARS-CoV-2 S with Cobas e601 analyzer (Roche Diagnostics, Switzerland). The samples were processed according to the manufacturer’s instructions. The linear measurement range of the assay was 0.40–250 U/mL. Samples above 250 U/mL were diluted further (1:10, 1:100, and 1:1000) within the measurement range of the assay. The test result of <0.80 U/mL was considered negative.

The patient received a single dose of the BNT162b2 COVID-19 mRNA vaccine on 3 September 2021. The patient tested negative (0.4 U/mL) for anti-S antibodies before receiving the vaccination. After the vaccination, the anti-S antibody concentration was measured on days 7, 14, and 21. The concentration of anti-S antibodies on days 7 and 14 post-vaccination was 0.4 U/mL. On day 21 after administration of the vaccine, the anti-S antibodies were found at 10.57 U/mL. There were no injection-site specific and systemic adverse reactions that had been reported within 7 days after vaccine administration. On 25 September 2021, the patient received a second dose of the BNT162b2 COVID-19 mRNA vaccine. However, the patient tested positive for SARS-CoV-2 by reverse-transcriptase polymerase chain reaction (RT-PCR) shortly after vaccination.

## 3. Discussion

Protein S (PS) is a vitamin K-dependent plasma glycoprotein synthesized in hepatocytes, endothelial cells, and megakaryocytes [5,6]. PS normally circulates in the blood at a concentration of ~20–25 µg/mL (300–350 nmol/L); approximately 60% of PS is bound to complement component C4b-binding protein (C4BP), and the remaining 40% is a free form [5,6]. PS structure consists of an N-terminal Gla domain, a thrombin-sensitive region (TSR), four epidermal growth factor (EGF)-like domains, and the C-terminal sex hormone-binding globulin (SHBG)-like region which is comprised of two laminin G-type (LG) domains [5,6]. The most recognized function of PS is anticoagulant. The anticoagulant activity of PS is mediated either by direct interaction with procoagulant and their active form or indirect interaction with other anticoagulant proteins such as activated protein C (APC) and tissue factor pathway inhibitor (TFPI). In addition, PS also serves as an activating ligand for the TAM family of receptor tyrosine kinases (RTKs), particularly TYRO3 and MERTK [7]. Signaling through RTKs will limit the intensity and duration of immune response with at least two distinct mechanisms: (1) the inhibition of proinflammatory cytokines production and secretion and (2) enhancing the clearance of apoptotic cells by phagocytosis [8,9].

Deficiency of PS characterized by reduced activity of PS can be hereditary or acquired, and it poses a risk of deep vein thrombosis and ischemic stroke [1]. In SARS-CoV-2 infection, the PS deficiency has been linked to excessive blood clotting and immune hyperactivation conditions, resulting in severe COVID-19 cases [3]. The study of 91 hospitalized COVID-19 patients in Romania showed that the low activities of PS were associated with the severity and mortality of COVID-19 disease [2]. Another study of 19 critically ill COVID-19 patients in Wuhan, China demonstrated that the level of their natural anticoagulants including PS was below the normal range [4]. Recently, the SARS-CoV-2 papain-like protease (PL^pro^) has been hypothesized to contribute to the hypercoagulable and hyperinflammation states by cleaving the PS [10]. A highly conserved cleavage site LXGG motif of PL^pro^ is found in the first LG domain of PS [10]. Cleaving this site by SARS-CoV-2 PL^pro^ may significantly disrupt the interaction of PS with anticoagulant proteins such as APC and TFPI that are important for preventing clot formation [5,6]. Moreover, LG domains are required for PS binding to TAM receptors, and thus cleavage of this PS site may reduce TAM receptor signaling that plays an essential role in limiting excessive immune response [11].

The high antibody response induced by a single dose BNT162b2 mRNA COVID-19 vaccine has been previously reported [12]. In our patient, the seroconversion occurred on day 21 following a single dose of the BNT162b2 mRNA vaccine. This finding is concordant with previous reports that indicated the seroconversion rate was highest at 21 days after the first dose of the BNT162b2 mRNA vaccine [13,14]. However, the anti-S antibody level in our patient displayed a tendency to be lower than the median value of antibodies concentration found in infection-naïve individuals on day 21 post-vaccination [13,15]. Although the involvement of PS in the development of host humoral immune response remains unclear, previous studies have shown that PS signaling through TAM receptor (MERTK) on lymphocyte T cells plays a role in inducing proliferation, cytokines release, and differentiation into memory cells [16,17]. Since lymphocyte T cells are crucial for activation and differentiation of B cells into memory cells and involve several processes for enhancing antibody response such as affinity maturation and isotype switching, the impairment of T cell response is possibly found in PS-deficient conditions likely contributes to lower antibody response after vaccination.

Furthermore, our patient was on warfarin treatment when she received the vaccination. Apart from the anticoagulant activity, warfarin has been known for its immunomodulating action [18]. The daily dose of oral warfarin at 2 to 4 mg could inhibit inflammatory signal transduction, resulting in decreased IL-6 production from macrophages [18]. Warfarin also potentially inhibits MERTK activation, which has a role in macrophage survival by reducing PS activity [19]. Additionally, in vitro study has shown the impairment of T cells activation and proliferation in the presence of warfarin [20]. Considering the immunomodulating activities of warfarin as described above potentially impact antibody response, in the current case, the warfarin treatment needs to be considered as a factor that determines the antibody response after vaccination.

In conclusion, BNT162b2 mRNA vaccine administration is safe and able to mount antibody response after a single dose in a PS-deficient patient under warfarin treatment. The possibly reduced anti-S antibody level compared to the anti-S antibody concentration among healthy individuals that previously reported following administration first dose of the BNT162b2 mRNA vaccine has important implications given the increased risk of severe COVID-19 in PS-deficient patients. The coincidental SARS-CoV-2 infection and mRNA vaccination potentially evidenced the lower immunogenic response in a PS-deficient patient. These concurrent events limit us from gaining a more comprehensive understanding of the BNT162b2 mRNA vaccine immunogenicity in PS-deficient patients. Larger and longer follow-up studies are needed to conclusively determine the safety of and antibody response in PS-deficient patients following administration BNT162b2 mRNA vaccine and whether the warfarin treatment may impact antibody response after vaccination.

## Data Availability

The data used to support the findings of this study are included within the article.
